# Conscious Brain-to-Brain Communication in Humans Using Non-Invasive Technologies

**DOI:** 10.1371/journal.pone.0105225

**Published:** 2014-08-19

**Authors:** Carles Grau, Romuald Ginhoux, Alejandro Riera, Thanh Lam Nguyen, Hubert Chauvat, Michel Berg, Julià L. Amengual, Alvaro Pascual-Leone, Giulio Ruffini

**Affiliations:** 1 Starlab Barcelona, Barcelona, Spain; 2 Neurodynamics Laboratory, Department of Psychiatry and Clinical Psychobiology, Psychology and Medicine Faculties, University of Barcelona, Barcelona, Spain; 3 Axilum Robotics, Strasbourg, France; 4 Neuroelectrics Barcelona, Barcelona, Spain; 5 Cognition and Brain Plasticity Unit, Department of Basic Psychology, University of Barcelona, Barcelona, Spain; 6 Berenson Allen Center for Noninvasive Brain Stimulation, Beth Israel Deaconess Medical Center, Harvard Medical School, Boston, Massachusetts, United States of America; Duke University, United States of America

## Abstract

Human sensory and motor systems provide the natural means for the exchange of information between individuals, and, hence, the basis for human civilization. The recent development of brain-computer interfaces (BCI) has provided an important element for the creation of brain-to-brain communication systems, and precise brain stimulation techniques are now available for the realization of non-invasive computer-brain interfaces (CBI). These technologies, BCI and CBI, can be combined to realize the vision of non-invasive, computer-mediated brain-to-brain (B2B) communication between subjects (*hyperinteraction*). Here we demonstrate the conscious transmission of information between human brains through the intact scalp and without intervention of motor or peripheral sensory systems. Pseudo-random binary streams encoding words were transmitted between the minds of emitter and receiver subjects separated by great distances, representing the realization of the first human brain-to-brain interface. In a series of experiments, we established internet-mediated B2B communication by combining a BCI based on voluntary motor imagery-controlled electroencephalographic (EEG) changes with a CBI inducing the conscious perception of phosphenes (light flashes) through neuronavigated, robotized transcranial magnetic stimulation (TMS), with special care taken to block sensory (tactile, visual or auditory) cues. Our results provide a critical proof-of-principle demonstration for the development of conscious B2B communication technologies. More fully developed, related implementations will open new research venues in cognitive, social and clinical neuroscience and the scientific study of consciousness. We envision that hyperinteraction technologies will eventually have a profound impact on the social structure of our civilization and raise important ethical issues.

## Introduction

**Figure 1:**
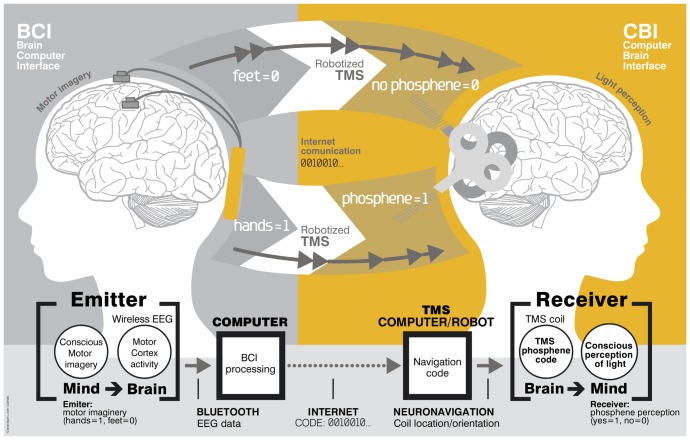
Brain-to-brain (B2B) communication system overview. On the left, the BCI subsystem is shown schematically, including electrodes over the motor cortex and the EEG amplifier/transmitter wireless box in the cap. Motor imagery of the feet codes the bit value 0, of the hands codes bit value 1. On the right, the CBI system is illustrated, highlighting the role of coil orientation for encoding the two bit values. Communication between the BCI and CBI components is mediated by the internet.

The evolution of civilization points to a progressive increase of the interrelations between human minds, where by “mind” we mean a set of processes carried out by the brain [1]. Until recently, the exchange of communication between minds or brains of different individuals has been supported and constrained by the sensorial and motor arsenals of our body. However, there is now the possibility of a new era in which brains will dialogue in a more direct way [2]. Previous attempts to realize this vision include demonstrations of bidirectional computer-brain communication [3]–[5] and cortical-spinal communication [6] in the monkey, and hippocampus-to-hippocampus [7] or social communication [8] in the rat – all of invasive nature. Despite these and other significant advances with human subjects [9]–[10], invasive methods in humans remain severely limited in their practical usefulness. Pioneering research in the 60's using non-invasive means already demonstrated the voluntary control of alpha rhythm de-synchronization to send messages based on Morse code [11]. Over the last 15 years, technologies for non-invasive transmission of information from brains to computers have developed considerably, and today brain-computer interfaces embody a well-established, innovative field of study with many potential applications [12]–[16]. Recent work has demonstrated fully non-invasive human to rat B2B communication by combining motor imagery driven EEG in humans on the BCI side with ultrasound brain stimulation on the CBI-rat side [17]. However, the realization of non-invasive CBI in humans remains elusive, and adequate methodologies to provide computer-mediated non-invasive brain conscious interventions are lacking. Here we show how to link two human minds *directly* by integrating two neurotechnologies – BCI and CBI –, fulfilling three important conditions, namely a) being non-invasive, b) cortically based, and c) consciously driven (Fig. 1). In this framework we provide the first demonstration of non-invasive direct communication between human minds.

## Materials and Methods

### Human Subjects

Four healthy participants (age range 28–50) were recruited, and their informed written consent was obtained. Of the four subjects, one was assigned to the BCI branch (the *emitter* - Subject 1) and the other three to the CBI branch of the experiments (i.e., as *receivers* - Subjects 2, 3 and 4).

### Ethics Statement

The Ethics Committee of the University of Barcelona, following the Ethical Principles for Medical Research Involving Human Subjects of the WMA Declaration of Helsinki, approved this study. The TMS part of the experiments was conducted according to TMS safety guidelines [18]. The individuals in this manuscript gave their written informed consent (as outlined in the PLOS consent form) to publish these case details.

### Methods Summary

The computer-mediated brain-to-brain transmission from Thiruvananthapuram (Kerala state, India) (BCI side) to Strasbourg, France (CBI) was realized using internet-linked EEG and TMS technologies respectively. On the CBI side, three information *receiver* subjects were stimulated with biphasic TMS pulses at a subject-specific occipital cortex site. The intensity of pulses was adjusted for each subject so that a) one particular orientation of the TMS-induced electric field produced phosphenes [19] (representing the “active direction” and coding the bit value “1”), and b) the orthogonal direction did not produce phosphenes (representing the “silent direction” and coding the bit value “0”). Subjects reported verbally whether or not they perceived phosphenes on stimulation. A fourth subject acted as *emitter* of information using a BCI system based on motor imagery (of moving feet or hands) to select two kinds of states in EEG spectral power in the motor cortex (coding for the bit values of “0” and “1”). We ensured that receiver subjects were not relying on peripheral nervous system (PNS) cues (visual, tactile and auditory sensations produced by the TMS device) to decode the information by blocking sensory cues: we used a force sensor on the coil to maintain a constant contact pressure on the scalp, implemented a coil rotation information encoding strategy (as opposed to one relying on coil location), and had subjects wear eye mask and earplugs. We verified the effectiveness of these means in series of *d-prime* control experiments [20]–[22] comparing pairs of stimuli delivered either with the same or different orientations of the coil. Finally, as performance measures for the BCI, CBI and B2B system we analyzed error transmission rates and transmission speed (bits per minute).

### Brain-Computer Interface

**Figure 2:**
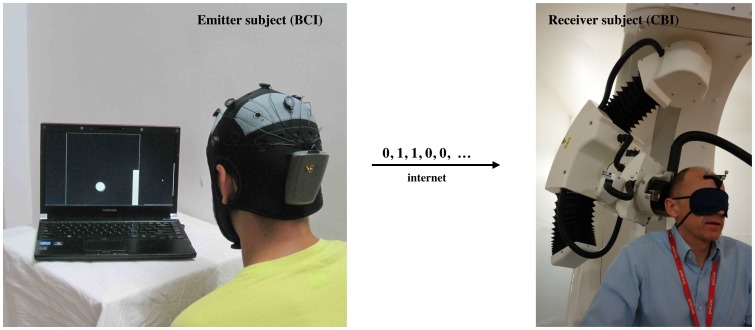
View of emitter and receiver subjects with non-invasive devices supporting, respectively, the BCI based on EEG changes driven by motor imagery (left) and the CBI based on the reception of phosphenes elicited by a neuronavigated TMS (right) components of the B2B transmission system. The successfully transmitted code in the particular scenario shown is a ‘0’: the target and ball are at the bottom of the screen (correctly encoding a 0 through motor imagery of the feet) and the TMS coil is in the orientation not producing phosphenes for this particular participant (subject 2, see Figure 3), with the handle pointing upwards.

The BCI communication subsystem used in our experiments converted conscious voluntary motor imagery into brain activity changes that could be captured non-invasively as physical signals conveying information. To monitor EEG activity related with motor imagery tasks we used a wireless (500 S/s, 24 bit) EEG recording system [23] (Starstim tCS/EEG system, by Neuroelectrics, http://www.neuroelectrics.com). Eight Ag/AgCl electrodes were placed at F3, F4, T7, C3, Cz, C4, T8 and Fz scalp sites (10–20 EEG positioning system) and electrically referenced to a clip electrode placed in the right ear lobe. A spatial filter was applied to the electrodes of interest (C3, Cz and C4) by referencing them to the average potential of their neighboring electrodes. To transform EEG signals into binary information we used the BCI-2000 platform [24] implementing the detection of anatomically localized changes in EEG related with voluntary motor imagery. The emitter subject was sequentially shown on the screen a representation of the bits to be transmitted (the message). Each bit was represented either by a target cue in the downright part of the screen (bit value 0) or in the upright part (bit value 1) (Figs. 1 and 2). If the bit to be transmitted was a 1 (0), the emitter was to encode it through motor imagery of the hands (feet). These motor imagery tasks controlled the vertical movement of a ball appearing on the screen from the left with a constant horizontal speed. If the ball hit the displayed target on the right of the screen, the transmitted bit was then correctly encoded. Whatever the outcome the BCI encoded bits were then automatically sent via email to the CBI subsystem. Following a training period, the emitter subject was able to regularly achieve an accuracy of well over 90% in BCI encoding.

### Computer-Brain Interface

**Figure 3:**
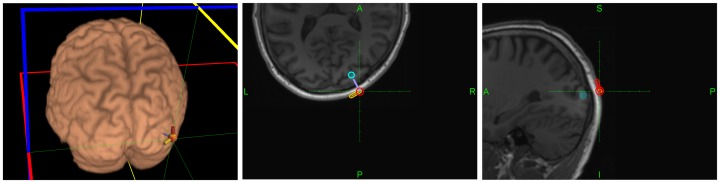
Location and orientation of hot spot for phospene production overlaid on MRI image of the head of subject 2 (see Figure 2). The active direction producing phospenes is highlighted in orange (in red, the orthogonal direction not producing phosphenes).

For the CBI subsystem, we relied on biphasic TMS pulses to encode information. For each receiver subject, we identified first a TMS phosphene-producing hotspot in the right visual occipital cortex (approximately 2 cm anterior and 2 cm right from inion, the precise location depending on the subject), which was used for the *active* condition (to encode the bit value ‘1’). We achieved the required high precision in relocation and reorientation of the TMS target by using a neuronavigated [25]–[28], robotized TMS system (Axilum Robotics TMS-Robot, http://www.axilumrobotics.com, piloted by Localite 2.8 Neuronavigation system using the MagVenture MagPro R30 TMS Stimulator with a “butterfly” coil of type Cool-B65-RO). Subjects went through a familiarization period in which we administered several TMS pulses to the chosen right occipital cortex site using various rotations of the coil, and identified the intensity of TMS pulses (range 57–90% of maximum intensity of the coil) that optimally discriminated *active* (i.e., producing phosphenes) from *silent* (not producing phosphenes) orientations (Fig. 3). Subjects described the sensations of light produced by TMS pulses of the active orientation as having a strong, clear and reliable nature, and located at the bottom of the visual field contralateral to the stimulation site [29]. They were instructed to report verbally the presence of phosphenes immediately after TMS pulse delivery. TMS pulses were administered by the robotized TMS system controlled by a researcher sitting away from the visual field of the subject, or directly programmed into the neuronavigation computer by the BCI message sequence received via email (Fig 2). Sequences of two or three redundant TMS pulses were delivered with an inter-stimulus interval of 2 seconds.

Our first robotized CBI experiments (subject 1) used a position-dependent encoding with the TMS hotspot representing the active condition (bit  = 1) and another scalp location (displaced about 2 cm from the first) representing the silent condition (bit = 0). This strategy was used for CBI transmissions of 60 bit messages with a low error rate. An associated first B2B experiment (Barcelona to Strasbourg) – carried out offline (i.e., with the BCI and CBI branches of transmission separated in time by buffering the data after BCI transmission) – resulted in a 15% transmission error rate (5% in the BCI segment and 11% in the CBI one). However, we identified the possibility that the receiver subject at the CBI end was being cued on the (active or silent) stimulation condition by PNS sensory inputs (tactile, auditory or visual) related to the repositioning of the coil at different scalp sites. In order to rule this out, we implemented a series of measures on the next experiments – including the final B2B transmissions described below. First, to avoid contact related cues and taking advantage of the anisotropic response of the visual cortex to TMS [30], we adopted the strategy of encoding bits through rotation of the TMS coil: the location and “active” orientation of the coil (producing phosphenes in most trials) were chosen with the condition that a 90° rotation of the coil on the same location did not produce phosphenes (Fig. 2). The robot was programmed to move the coil away from the scalp after the delivery of each triad of TMS pulses. A force sensor on the coil surface was used to maintain a constant contact force with the scalp in all conditions. The cable holder on the robot was adjusted to keep the coil's cable at a good distance from the subject's shoulders and back, preventing contact during coil rotation. Second, to avoid identification of coil orientation from auditory information, subjects wore earplugs and the robot moved the coil between each pair or triads of TMS pulses towards a parking site located approximately 1 cm away from the scalp with an intermediate rotation of 45°. This forced the robot to realize a movement of similar duration and with equal noise levels for all bit transmission events, irrespective of coil orientation. Lastly, we blocked visual cues on stimulation configuration by having subjects close their eyes and wear an eye mask.

To assess the effectiveness of these measures, we carried out a series of control studies using the sensitivity index (or *d-prime*) statistic [20]–[22]. The first control studied TMS noise induced auditory cueing and had subjects (2 and 3) wear an eye mask and earplugs and receive a sequence of 32 balanced pairs of three TMS stimuli randomly interspersed over silent and active conditions. We mimicked the contact of the coil but eliminated the production of phosphenes by interposing, between coil and scalp, a single piece of foam slightly displacing (∼1 cm) the center of the coil orthogonally away from the head. After the administration of each pair (of triads) of stimuli, subjects were asked if they were delivered with the equal or different orientations. Then, we performed a second control experiment to evaluate cues from (tactile) skin contact, based on another sequence of 32 balanced pairs, without foam on the coil but setting a null intensity in the magnetic stimulator. Results from these tests indicated with high confidence that, after correct blinding of auditory, visual or tactile cues, the subjects were unable to distinguish coil orientations in the absence of actual phosphene-inducing TMS pulses (Subject 2: d′ = 0.0 in the auditory task, d′ = −0.1 in the skin contact task; Subject 3: d′ = 0.6 in the auditory task, d′ = 0.1 in the skin task).

## Results

The final round of experiments targeted the demonstration of online brain-to-brain transmission of information between remotely located subjects. On March 28^th^, 2014, 140 bits were encoded by the BCI emitter in Thiruvananthapuram and automatically sent via email to Strasbourg, where the CBI receiver (subject 3) was located. There, a program parsed incoming emails to navigate the robot and deliver TMS pulses precisely over the selected site and with the appropriate coil orientation. A similar transmission with receiver subject 2 took place on April 7^th^, 2014. In both cases, the transmitted pseudo-random sequences carried encrypted messages encoding a word – “*hola*” (“*hello*” in Catalan or Spanish) in the first transmission, “*ciao*” (“hello” or “goodbye” in Italian) in the second. Words were encoded using a 5-bit Bacon cipher [31] (employing 20 bits) and replicated for redundancy 7 times (for a total of 140 bits). The resulting bit streams were then randomized using random cyphers selected to produce balanced pseudo-random sequences of 0's and 1's (for subject blinding and proper statistical analysis purposes in addition to providing word-coding). On reception, de-cyphering and majority voting from the copies of the word were used to decode the message.

In these experiments, the individual BCI and CBI segments as well as the complete B2B link provided transmission of pseudo-random information with excellent integrity. In the first experiment the transmission error rates were of 6%, 5% and 11% for the BCI, CBI and the combined B2B components respectively, and in the second, error rates were of 2%, 1% and 4% respectively. We note that the probability of transmission of lists of 140 items having occurred with the low observed error rates or less by chance is negligible (p<10^−22^). For example, the probability of guessing correctly 140 random, balanced bits with an error rate of 20% (28 errors out of 140) or less is extremely low, this being equivalent to obtaining 112 heads or more after 140 tosses of a fair coin (p<10^−13^).

BCI and CBI transmission rates were of 3 and 2 bits per minute respectively. The overall B2B transmission speed was of 2 bits per minute (limited by the CBI branch). The encoded words were transmitted with full integrity by all links – BCI, CBI and B2B.

## Discussion

In these experiments we demonstrated the feasibility of direct brain-to-brain communication in human subjects, with special care taken to ensure the non-participation of sensory or motor systems in the exchange of information (Figure 1). Streams of pseudo-random bits representing the words “*hola*” and “*ciao*” were successfully transmitted mind-to-mind between human subjects separated by a great distance, with a negligible probability of this happening by chance.

We believe these experiments represent an important first step in exploring the feasibility of complementing or bypassing traditional language-based or other motor/PNS mediated means in interpersonal communication. Although certainly limited in nature (e.g., the bit rates achieved in our experiments were modest even by current BCI standards, mostly due to the dynamics of the precise CBI implementation), these initial results suggest new research directions, including the non-invasive direct transmission of emotions and feelings or the possibility of sense synthesis in humans, that is, the direct interface of arbitrary sensors with the human brain using brain stimulation, as previously demonstrated in animals with invasive methods [2].

The main differences of this work relative to previous brain-to brain research are a) the use of human emitter and receiver subjects, b) the use of fully non-invasive technology and c) the conscious nature of the communicated content. Indeed, we may use the term *mind-to-mind* transmission here as opposed to *brain-to-brain*, because both the origin and the destination of the communication involved the conscious activity of the subjects.

Our findings strengthen the relevance of integrating the CBI branch in human-computer communication using precision technologies for high performance (i.e., a robotized, neuronavigated TMS system). Importantly, we demonstrated the use of rotation-encoding TMS induced phosphenes as a reliable CBI solution, providing methods and controls to exclude PNS involvement.

The proposed technology could be extended to support a bi-directional dialogue between two or more mind/brains (namely, by the integration of EEG and TMS systems in each subject). In addition, we speculate that future research could explore the use of closed mind-loops in which information associated to voluntary activity from a brain area or network is captured and, after adequate external processing, used to control other brain elements in the same subject. This approach could lead to conscious synthetically mediated modulation of phenomena best detected subjectively by the subject, including emotions, pain and psychotic, depressive or obsessive-compulsive thoughts.

Finally, we anticipate that computers in the not-so-distant future will interact directly with the human brain in a fluent manner, supporting both computer- and brain-to-brain communication routinely. The widespread use of human brain-to-brain technologically mediated communication will create novel possibilities for human interrelation with broad social implications that will require new ethical and legislative responses [32].

## Supporting Information

Info S1 **Provides details on TMS procedures, BCI and PNS control experiments.**
(DOC)

Data S1 **Contains data from experiments involving BCI and B2B transmissions as well as in d-prime paired tests.**
(ZIP)
