# Supplementary material for: Conscious Brain-to-Brain Communication in Humans Using Non-Invasive Technologies
Source: PLoS One. 2014 Aug 19;9(8):e105225. doi: 10.1371/journal.pone.0105225 (PMC4138179; doi:10.1371/journal.pone.0105225)
Supplement: Info S1 — Provides details on TMS procedures, BCI and PNS control experiments. (DOC) [file pone.0105225.s001.doc]

**S1- Supplementary Information**

**for *Conscious Brain-to-Brain Communication in Humans Using Non-Invasive Technologies, Grau et al., 2014.***

Cipher used for word transmission:

To encode the 4-letter words we used a Bacon cipher (Matlab code follows):

%% First we encode the letters using the Bacon cypher:

h = [0 0 1 1 1];

o = [0 1 1 0 1];

l = [0 1 0 1 0];

a = [0 0 0 0 0];

% now we form the word bit string:

word=[ h o l a ];

% and the resulting strings repeated 7 times,

Reps=7;

repword=repmat(word,1,Reps);

%% Encrypting and balancing (for later stat analysis)

% Repeat this block till you get a sum of 50% of the length for the sum.

% First, create the randomizing cipher

cypher = round(rand(length(repword),1)');

% Encrypt the word

transmit= mod(cypher+repword,2);

% check that we have 50% 1s and 0s:

sum(transmit) % should be 70=?10/2

%check we can decode after reception! This shoudl be zero:

sum(repword - mod(transmit+cypher,2))

%% Summarizing.

% Message to transmit:

transmit

% decoding cipher

cypher

E.g, the second 140 string transmitted was

transmit= [1 1 0 0 1 0 1 1 1 1 0 1 1 0 1 0 1 0 1 0 1 0 1 0 1 1 0 0 1 0 1 0 0 0 0 1 0 1 1 1 0 0 1 0 0 0 1 1 1 1 0 1 1 0 0 0 0 1 1 1 1 0 1 0 11 0 0 0 0 1 0 1 0 1 0 1 1 1 1 0 1 1 0 1 0 0 1 0 1 0 0 1 1 1 0 0 1 0 1 0 1 0 0 0 1 1 1 1 1 0 0 0 1 0 1 0 0 0 1 0 1 0 0 1 1 1 0 0 1 0 1 0 0 0 0 0 0 1 1].

Decoding was done again by addition of the cipher mod 2. The resulting 140 bit string was then split into 7 blocks, and each block into 4 letters of 5 bits each. Majority voting from each block was used to fix the final bit value for the 20 bit encoded word.

Excel files with the analysis of BCI and CBI transmission are included as supplementary information files.

Statistical analysis of transmissions.

As noted in our paper, the probability of transmission of lists of 140 balanced items having occurred with the very low observed error rates or less by chance is negligible (p< 10^-22^). For example, the probability of guessing correctly 140 random, balanced bits with an error rate of 20% (28 errors out of 140) or less is extremely low (equivalent to obtaining 112 heads or more after 140 tosses of a fair coin, p<10^-13^).

d-prime analysis/control tests

Data regarding these tests is provided in Excel files in Supplementary Data S2 zipped file.

TMS operation

Regarding deprivation of light and vision with the eye mask: 5 minutes before the start of the B2B transmissions. And subjects were about four times for 15 min in the same conditions as familiarization and training before conducting the real B2B.

BCI operation

The BCI side of the experiment took place in Kazhakootam, a municipality belonging to Trivandrum, the capital of Kerala, a southern state of India.

The BCI emitter subject was a well-trained BCI user. The 140 bit encoded word was input in BCI2000 (1 coding the upper target and 0 coding the lower target). The 140 code was transmitted in chunks of 20 (+1) bits to allow the subject to rest between BCI runs. In each run, the first bit was used for BCI calibration purposes and was discarded from the transmission. So for each hyperinteraction experiment, the subject transmitted 20 bits in 7 different runs.

One computer was used to perform the BCI sessions, providing the necessary visual feedback for the proper BCI functioning. Another computer was used to automatically send via email the BCI coded bits (one by one) using an automatic mailing system program that was developed for this purpose.

The theoretical time for the transmission can be found in the table below:


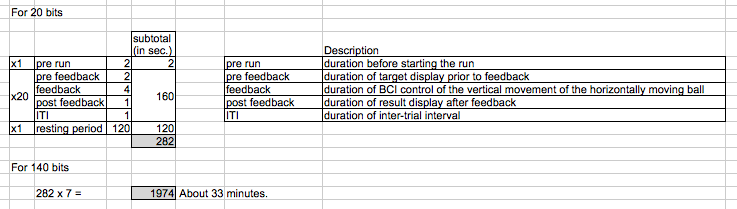


It actually took a little bit longer in the real experiments (about 45 minutes) due to longer resting periods between BCI runs.

BCI data samples (in BCI2000 data format) with 100% hits, an excel file with BCI annotations can be found in the provided BCI folders in the **S2 – Supplementary data** zipped file.
